# Supplementary material for: Versatile Product Detection via Coupled Assays for Ultrahigh-Throughput Screening of Carbohydrate-Active Enzymes in Microfluidic Droplets
Source: ACS Catal. 2023 Jul 21;13(15):10232–43. doi: 10.1021/acscatal.3c01609 (PMC10407846; doi:10.1021/acscatal.3c01609)
Supplement: Supplementary file 1 — cs3c01609_si_001.pdf [file cs3c01609_si_001.pdf]

Supporting Information

Versatile Product Detection via Coupled Assays for Ultra-high-throughput Screening of Carbohydrate-Active-Enzymes in Microfluidic Droplets

Simon Ladeveze†, Paul J. Zurek, Tomasz S. Kaminski#, Stephane Emond and Florian Hollfelder*

Department of Biochemistry, University of Cambridge, 80 Tennis Court Road, Cambridge CB21GA, UK.

Corresponding Author

* F. Hollfelder (FH111@cam.ac.uk).

Present Address

† S. Ladeveze, TBI, CNRS, INRAE, INSAT, Université de Toulouse, F-31400, Toulouse, France. #T.S. Kaminski, Department of Environmental Microbiology and Biotechnology, Institute of Microbiology, Faculty of Biology, University of Warsaw, Miecznikowa 1, 02-096 Warsaw, Poland.

Table of Contents

1. Considerations regarding the choice of coupling enzymes and assay robustness: 1
2. Practical considerations regarding the sorting operations: 3

Supplementary Figure S1: 5
Supplementary Figure S2: 6
Supplementary Figure S3: 9
Supplementary Figure S4: 10
Supplementary Figure S5: 11

1. **Considerations regarding the choice of coupling enzymes and assay robustness:**

1.1. Various methods using coupling reactions are available for the detection of the different monosaccharides that are the focus of this study[1–4]. However, care is needed when choosing candidates that do not have promiscuous activities to ensure specific detection. For example, to monitor glucose, hexose oxidase has been reported in the literature, but this enzyme is not exclusively specific for glucose and recognize other monosaccharides[5]. This promiscuity can produce false positives when assaying a substrate containing other hexoses, *e.g.* mannose in glucomannan or xylose in xyloglucan. Glucokinase by contrast is a suitable alternative because it is very specific for glucose [6]. However, the detection of glucose then requires a two-step cascade, as this enzyme uses ATP to convert glucose to glucose-6-phosphate, which is subsequently converted to 6–phosphoglucono-γ-lactone by glucose-6-phosphate dehydrogenase, whilst generating NADPH[7]. As it is only composed of glucose units, cellulose is a good test case for this reaction cascade. However, in Nature, cellulose degradation is a complex process that involves multiple enzymes for which the product is not always glucose. Endo-acting cellulases for instance generate short chain cello-oligosaccharides, and cellobiohydrolases release cellobiose. Similarly to xylan degradation, these products are subsequently processed by β-glucosidases to release glucose. To release glucose from cellulose, Avicel, carboxymethylcellulose (CMC) and cellohexaose (G6) were used as substrates for a commercial GH5 cellulase supplemented, or not, with a GH3 β-glucosidase to yield glucose as final product, mimicking the natural cellulose degradation process. As shown on Supplementary Figure 2A, glucose could effectively be detected by the cascade reaction of glucokinase/glucose-6-phosphate dehydrogenase. Using the cellulase alone only released traces of glucose, probably due to the presence of trace amounts of shorter cello-oligosaccharides. As expected, the addition of β-1,4-glucosidase largely improved the glucose release, with an increase of absorbance values by 0.1 and 0.05 mM for CMC and Avicel, respectively. These results validated the use of this enzymatic cascade to assay both cellulase and β-glucosidase activities.

1.2. Beechwood xylan (BX) is formed of a main xylose chain branched with glucuronic acid side chains. Incubation of the GH67 α-glucuronidase with arabinoxylan (WAX) or beechwood xylan (BX) did not result in the release of glucuronic acid (Supplementary Figure 2B). This may be due to two reasons: i) xylan from beechwood only contains a small (<10%) amount of α-1,2 linked glucuronic acids, mainly C_6-metylated and ii) this commercial α-glucuronidase from *Bacillus stearothermophilus* has been previously reported to have no activity on intact xylan[8]. A High-purity aldouronic acids mixture (Megazyme reference O-AMXR), which mimics the substructure of beechwood xylan at branching points, was on the contrary a suitable substrate which allowed the release of glucuronic acid. As this substrate is only formed of short chains xylo-oligosaccharides (Degree of Polymerization (DP) <4) no synergistic effect could be observed upon addition of β-xylosidase. This model substrate therefore appears to be the most appropriate substrate for α-glucuronidase activity screening, as some α-glucuronidases can only accommodate xylan fragments, or xylans without methylations, which would not release glucuronic acid when using beechwood xylan.

1.3. Wheat arabinoxylan (WAX) is formed of a xylose main chain, with 38% α-1,2/α-1,3 linked arabinose ramifications. In nature, debranching enzymes such as GH51 α-arabinofuranosidases (ara*f*ases) act by removing the branches, releasing arabinose

monomers, along with a naked xylan chain. This substrate was used to detect arabinose. The detection was achieved using galactose dehydrogenase on arabinose released by a debranching ara*f*ase. Galactose dehydrogenase is both active on galactose and arabinose and oxidizes them to D-galactonic acid, and D-arabinonic acid respectively, whilst releasing NADH. We used a GH51 arafase from *Cellvibrio japonicus*, which can remove arabinoses branched with both α-1,2/α-1,3 linkages[9]. Incubation of ara*f*ase with WAX allowed the detection of a modest 0.1 mM arabinose release over 1h at 25°C, consecutively to the oxidation of the released arabinose monomers (Supplementary Figure 2C). However, the addition of xylanase increased this value up to 0.45 mM, demonstrating that this ara*f*ase is more active on shorter xylan fragments. As expected, when assayed on beechwood xylan, no absorbance signal could be detected in any condition, as this substrate is devoid of such arabinose ramifications, with the notable exception of a slight signal in the presence of β-xylosidase. This is probably due to the presence of 4.9% contaminating sugars in the commercial beechwood xylan solution, which might be short-chain arabinoxylan contaminants that are not degraded by xylanase (probably due to steric hindrance) whilst being too long to be processed by the ara*f*ase.

1.4. We used xylose dehydrogenase to convert xylose to xylonic acid and NADH. In nature, endo-xylanases generate short-chain xylooligosaccharides (XOS) from xylans that are further degraded to xylose by β-xylosidases. Here, we used a commercial xylanase formed by the catalytic domain of Xyn11A from *Neocallimastix patriciarum*. Contrarily to many xylanases, this recombinant enzyme can release a relatively small amount of xylose in addition to the main product xylobiose, releasing 0.39 and 0.45 mM xylose (Supplementary Figure 2D). As expected, on all substrates tested, the addition of the natural partner β-xylosidase largely improved the xylose release, with values increasing to 1.56 and 1.17 mM when assayed on beechwood xylan and WAX, respectively. As a consequence, for a general xylanase functional screening, β-xylosidase should be systematically added to the coupling reaction mix to ensure the proper release of xylose. If one is focusing on screening β-xylosidase activity, purified xylobiose appeared as a suitable substrate for the screening, as it released 1.8 mM xylose. Supplementation of xylanase and xylosidase with the corresponding debranching enzymes (*i.e.* glucuronidase and ara*f*ase, for BX and WAX, respectively) also produced a synergistic effect on total xylose release, increasing the xylose release by 0.49 mM, to 2.04 mM and by 0.12 mM to 1.29 mM, respectively. On the contrary, in the case of the aldouronic acid mixture, when used without β-xylosidase, the xylanase alone could not release higher amounts of xylose because it cannot efficiently convert DP <3, even with the added β-glucuronidase. This synergistic effect could only be observed when β-xylosidase was incubated with this substrate.

2. **Practical considerations regarding the droplet sorting operations**.

Automatic peak detection using LabVIEW is based on the live analysis of voltage values obtained from the absorbance detector. The program is identifying droplets by detecting a transient change in the raw trace, in the form of a signal dropping below the baseline (input value in the program). It identifies droplet edges and extracts each droplet's voltage (*i.e* droplets absorbance) as the value at the center of the droplet, or as the lowest value in this window (Supplementary Figure S5A & C, respectively). This requires the use of an absorbance offset, as the voltage at the center of the droplet may be above the baseline, even though the signal

corresponding to the droplet edges is below the baseline. This phenomenon causes the automatic detection program to fail (Supplementary Figure S5B). The program can also incorrectly extract the droplet voltage values due to the signal change at the droplet edges, which can be lower than the value at the center of the droplets (Supplementary Figure S5C). In such case a refractive index modifier is used to linearize the signal change when the laser is at the interface of the droplet with the oil (Supplementary Figure S5D). Note that this compound makes the droplets less visible in direct light when setting up the experiment, and the actual concentration used should be adjusted to each carrier phase as all fluorinated oils do not have the same refractive index.

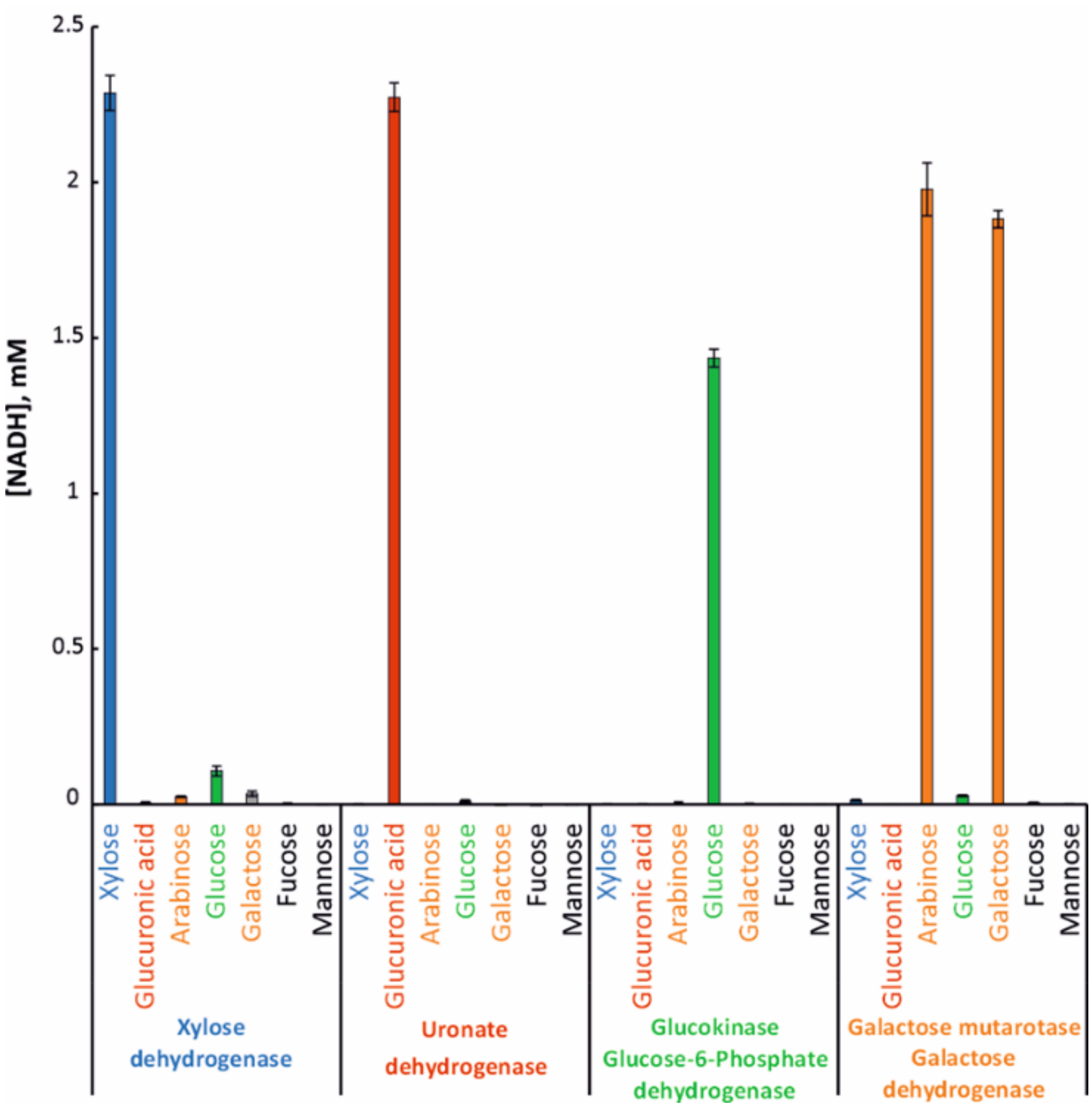

Supplementary Figure S1: Specificity of the chosen coupling reactions. Each coupling enzyme or enzymatic cascade was assayed against various monosaccharides, and activity was determined relative to the monosaccharide with the highest activity. Each coupling enzyme or enzymatic cascade (glucokinase + glucose-6-phosphate dehydrogenase) has been tested on 7 monosaccharides (x-axis). The vertical bars indicate the amount of released NADH after 1h of incubation of the cascade with the carbohydrates. Colors match the enzymatic cascades with the targeted monosaccharides. All enzymes were specific for their substrate, with <2% of activity on all other monosaccharides, with the notable exception of glucose with xylose dehydrogenase (4.7% the activity on xylose) and galactose dehydrogenase (100% and 95% on arabinose and galactose, respectively).

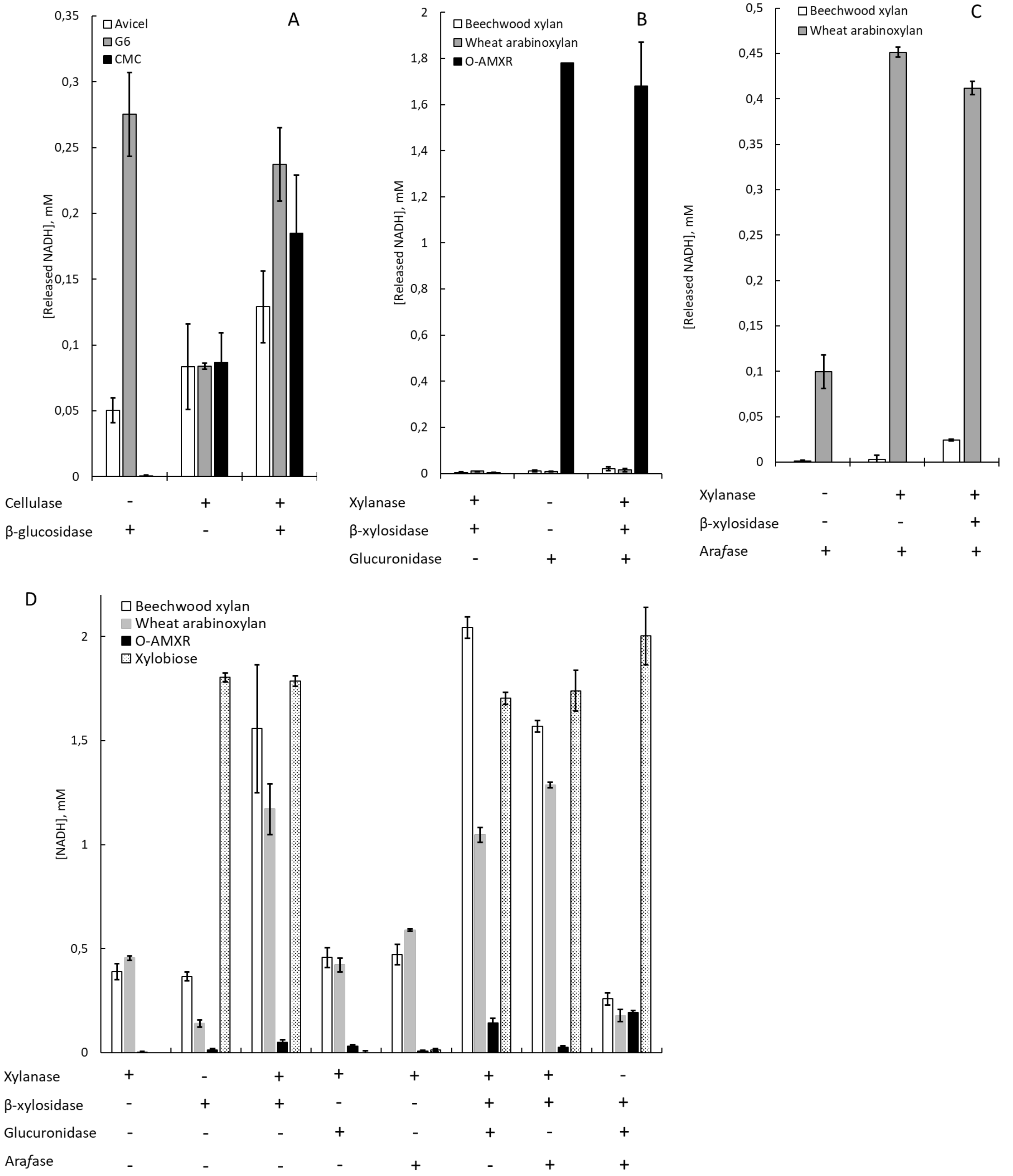

Supplementary Figure S2: Analysis of the specificity of coupling reactions using natural substrates with purified enzymes. The product concentrations were measured by NADH (or NADPH)-release by coupling enzymes. (A) Glucose release from Avicel (white), CMC (black) or

cellohexaose (white) by GH5 endo-cellulase supplemented or not by GH3 β-glucosidase (*A. niger*). B) Glucuronic acid release from Beechwood xylan (white), wheat arabinoxylan (grey) and aldouronic acids (black) by GH67 glucuronidase supplemented or not by GH11 endo-xylanase and GH43 β-xylosidase. C) Arabinose release from beechwood xylan (white) or wheat arabinoxylan (grey) by GH51 Arabinofuranosidase supplemented or not by GH11 endo-xylanase and/or GH43 β-xylosidase. D) Xylose release from Beechwood xylan (white), wheat arabinoxylan (grey), aldouronic acids (black) or xylobiose (dots) by GH11 endo-xylanase supplemented or not by GH43 β-xylosidase, GH67 α-glucuronidase or GH51 Arafase. Error bars indicate the standard deviation from a triplicate experiment.

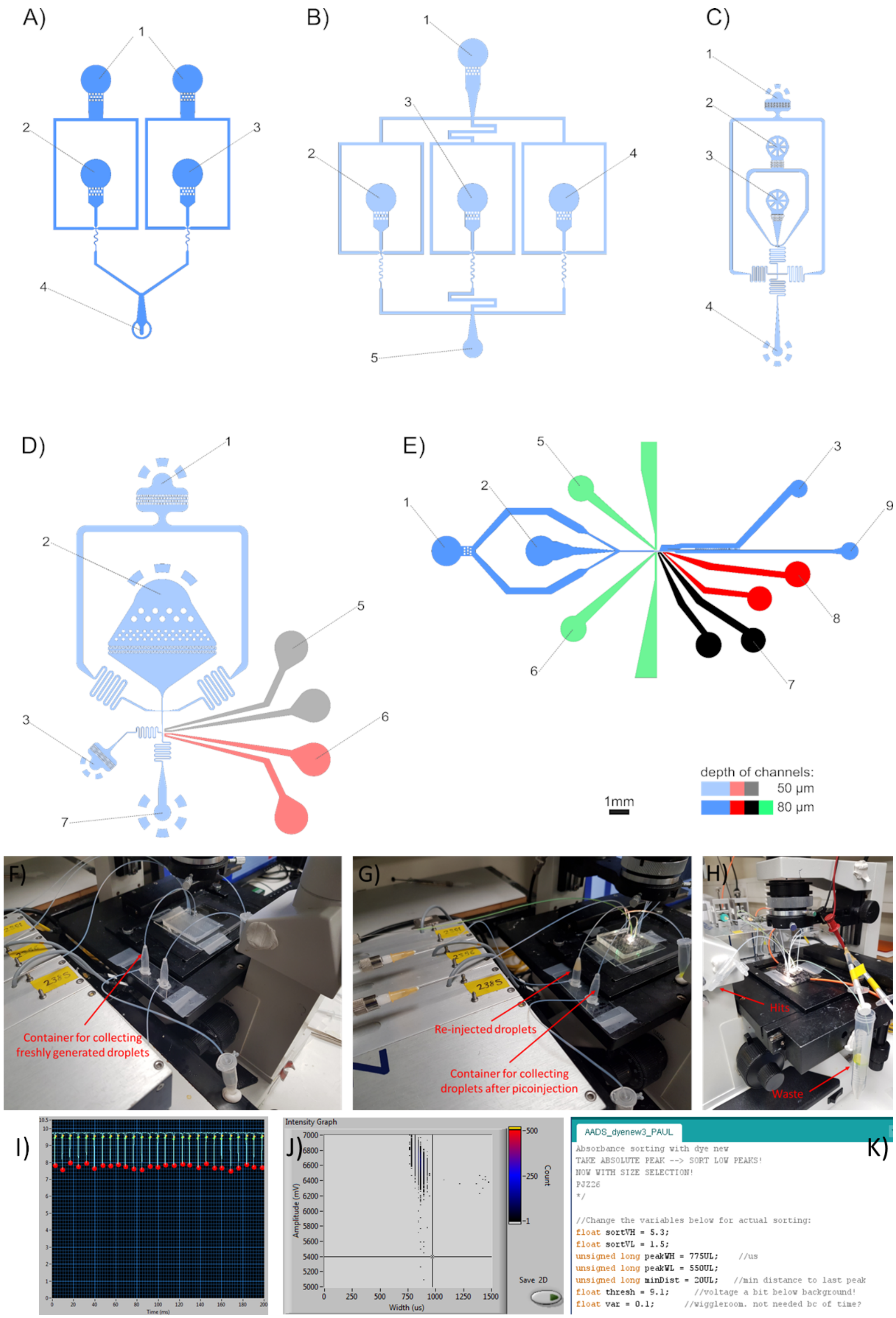
A)
B)
C)
D)
E)
depth of channels:
50 µm
80 µm
1mm
F)
Container for collecting
freshly generated droplets
G)
Re-injected droplets
Container for collecting
droplets after picoinjection
H)
Hits
Waste
I)
Time (ms)
J)
Intensity Graph
Amplitude (mV)
Width (us)
Count
Save 2D
K)
AADS_dyenew3_PAUL
Absorbance sorting with dye new
TAKE ABSOLUTE PEAK --> SORT LOW PEAKS!
NOW WITH SIZE SELECTION!
PJZ26
*/
//Change the variables below for actual sorting:
float sortVH = 5.3;
float sortVL = 1.5;
unsigned long peakWH = 775UL; //us
unsigned long peakWL = 550UL;
unsigned long minDist = 20UL; //min distance to last peak
float thresh = 9.1; //voltage a bit below background!
float var = 0.1; //wiggleroom. not needed bc of time?

Supplementary Figure S3: Chip designs used in this study. (A) Double flow-focusing droplet generator design for dual population analysis: (1) oil inlet, (2) and (3) aqueous phase inlets, 4) droplet outlet. (B) Triple flow-focusing droplet generator: (1) oil inlet, (2), (3) and (4) aqueous phase inlets, (5) droplet outlet. This chip's design is derived from the double flow-focusing device (A). The design has been optimized to generate the same oil pressure at each junction from one oil inlet. (C) Flow focusing droplet generator used in cell assays: (1) oil inlet, (2) fresh medium inlet; (3) cells inlets, (4) droplet outlet. (D) Pico-injector: (1) oil inlet, (2) droplet reinjection inlet, (3) inlets for picoinjection of solutions; (4) picoinjected droplets outlet, (5) negative electrode channel, (6) positive electrode channel. (E) Absorbance-Activated Droplet sorter: (1) oil inlet, 2) droplet reinjection inlet, (3) waste outlet, (4) positive sorted droplets, (5) & (6) optic fibers insertion channels, (7) negative electrode channel, 8) positive electrode channel. The separator between the positive and negative exit channels contains perforations to reduce backpressure. All chip designs and CAD files are available at https://openwetware.org/wiki/DropBase:Devices. The microfluidic system shown in (F) is fitted with the flow focusing droplet generator (design C) with home-made containers collecting the formed droplets. The syringes filled with oil, cells solution and fresh autoinducible media are actuated by the pump system on the left. In (G) the collected droplets are re-injected onto the picoinjector (design D), and picoinjected with the coupling reaction reagents (green solution). In (H) a sorting experiment is being conducted, with the "waste" and "hits" collected in separate tubes. Positive and negative electrodes are colored in red and black, respectively. (I) Raw trace of droplets analyzed by the absorbance detector and visualized in LabView (y-axis indicates voltage values). From this raw trace a 2D-plot distribution (J) allows easy visualization of the gates to be used for droplet sorting. These values can be adjusted live and manually fed into the Arduino's chip script (K). Six main parameters can be modified: FloatVH and FloatFL: the absorbance gate (upper and lower voltage values [*i.e* absorbance window of droplets to be sorted]); peakWH and peakWL: the droplet size gate (width of droplets to be sorted, deduced from the time needed to pass through the detector [excluding either too small or too large droplets or fusions]), minDist: the minimal distance between two consecutive droplets (used for removal of satellites), ,and thresh: the baseline value used for live automatic droplet detection.

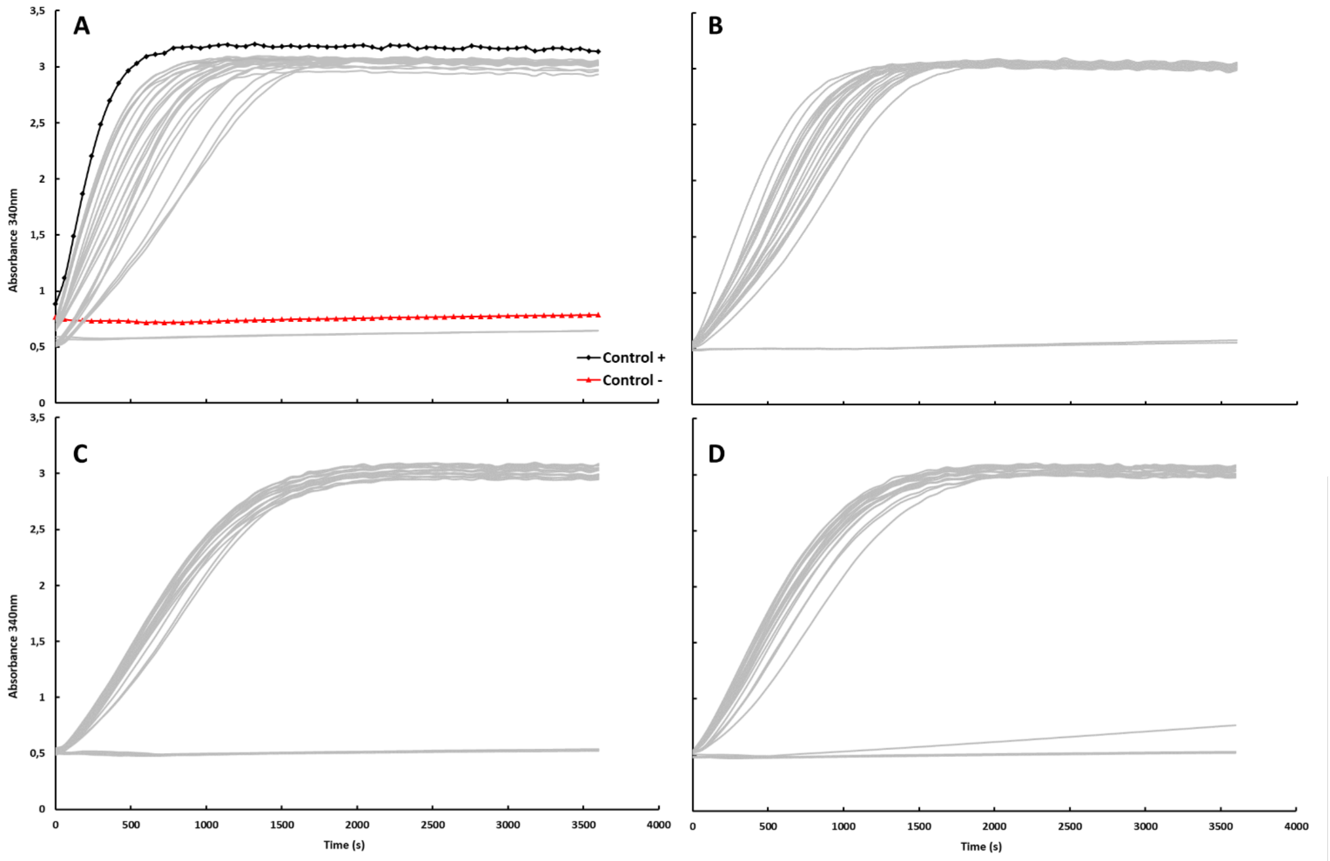

Supplementary Figure S4: Secondary screening of 96 randomly selected clones retrieved from β-xylosidase sorting. After sorting, purified DNA was transformed into *E. Coli* BL21 (DE3). Individual clones were cultured in autoinducible medium and culture supernatants were assayed for xylosidase activity using xylobiose as substrate. Xylose release was measured via NADH absorbance using the xylose detection cascade. Positive control: *Hi*Xyl43A expressing cells (black diamonds); negative control: *Sth*Araf62A expressing cells (red triangles). A) clones 1-24, B) clones 25-48, C) clones 49-72, D) clones 73-96. To take into account phenotypic variation, clones were considered positive when the absorbance signal reached saturation (plateau) in less than 2000 seconds under the conditions of the assay.

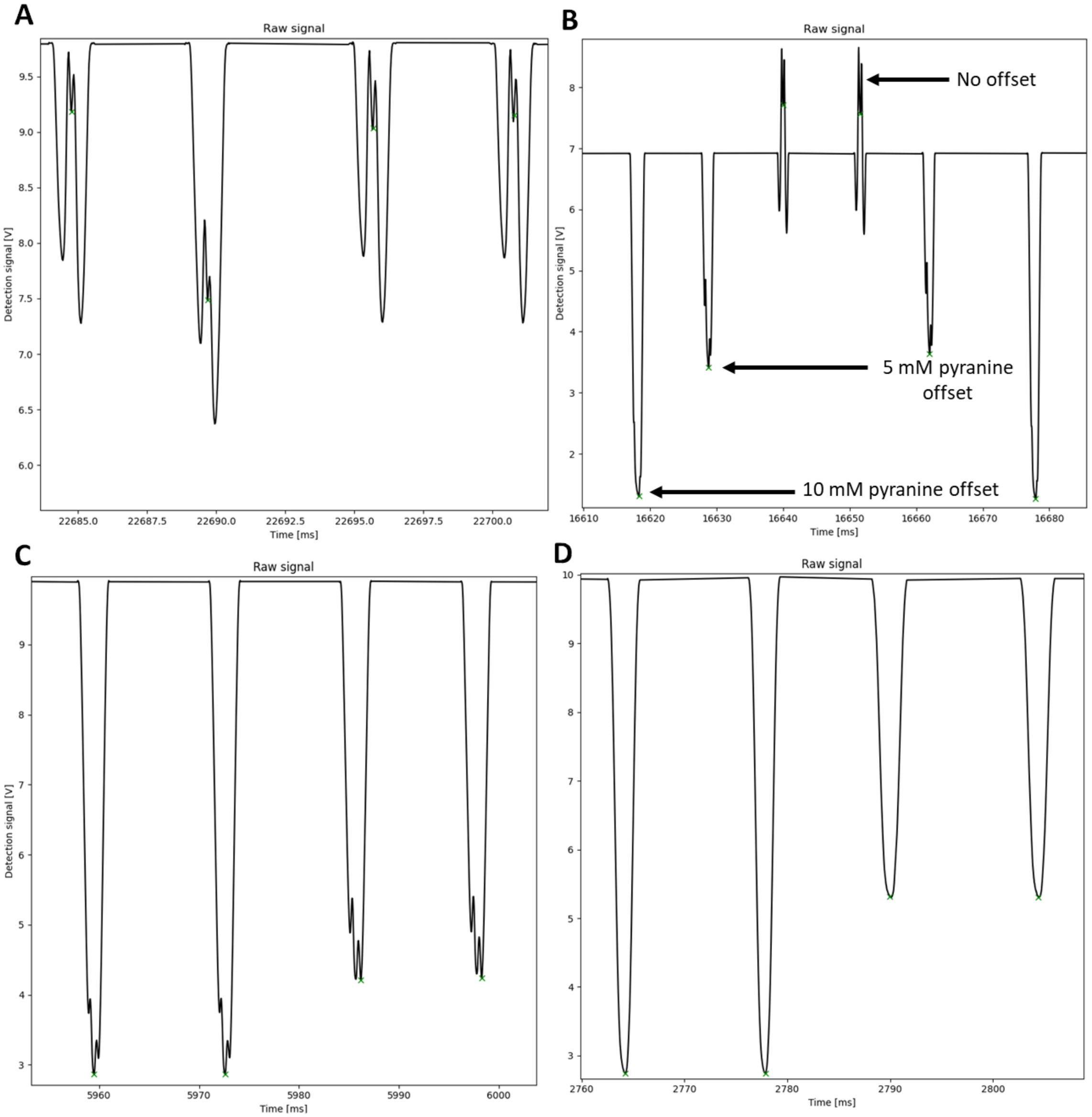

Supplementary Figure S5: A) Raw trace from the absorbance detector with peak detection (green crosses). The lowest voltage values are not at the center of the droplets and should not be used to measure droplet absorbance values. B) Effect of adding 5 mM or 10 mM pyranine absorbance offset to the signal. Five millimolar pyranine is sufficient to bring the signal below the baseline and ensure a proper peak detection. Raw trace without C) and with D) addition of 22.5% 1-Bromo-3,5-bis(trifluoromethyl)benzene. The droplet edges' signal is removed and droplets absorbances is easily extracted.

REFERENCES

(1) Scott, D. A.; Goward, C. R.; Scawen, M. D.; Atkinson, T.; Price, C. P. Colorimetric Glucose Assay Using Thermostable Glucokinase. *Ann. Clin. Biochem. Int. J. Lab. Med.* **1990**, *27* (1), 33–37. https://doi.org/10.1177/000456329002700107.

(2) Charnock, S. C.; McCleary, B. V. Assay for Determination of Free D-Galactose and/or L-Arabinose. US7785771B2.

(3) Yamanaka, K.; Gino, M.; Kaneda, R. A Specific NAD- D -Xylose Dehydrogenase from *Arthrobacter* Sp. *Agric. Biol. Chem.* **1977**, *41* (8), 1493–1499. https://doi.org/10.1080/00021369.1977.10862702.

(4) Pick, A.; Schmid, J.; Sieber, V. Characterization of Uronate Dehydrogenases Catalysing the Initial Step in an Oxidative Pathway. *Microb. Biotechnol.* **2015**, *8* (4), 633–643. https://doi.org/10.1111/1751-7915.12265.

(5) Ostafe, R.; Prodanovic, R.; Lloyd Ung, W.; Weitz, D. A.; Fischer, R. A High-Throughput Cellulase Screening System Based on Droplet Microfluidics. *Biomicrofluidics* **2014**, *8* (4), 041102. https://doi.org/10.1063/1.4886771.

(6) Meyer, D.; Schneider-Fresenius, C.; Horlacher, R.; Peist, R.; Boos, W. Molecular Characterization of Glucokinase from Escherichia Coli K-12. *J. Bacteriol.* **1997**, *179* (4), 1298–1306.

(7) Zhu, A.; Romero, R.; Petty, H. R. An Enzymatic Colorimetric Assay for Glucose-6-Phosphate. *Anal. Biochem.* **2011**, *419* (2), 266–270. https://doi.org/10.1016/j.ab.2011.08.037.

(8) Choi, I. D.; Kim, H. Y.; Choi, Y. J. Gene Cloning and Characterization of Alpha-Glucuronidase of Bacillus Stearothermophilus No. 236. *Biosci. Biotechnol. Biochem.* **2000**, *64* (12), 2530–2537.

(9) Beylot, M. H.; McKie, V. A.; Voragen, A. G.; Doeswijk-Voragen, C. H.; Gilbert, H. J. The Pseudomonas Cellulosa Glycoside Hydrolase Family 51 Arabinofuranosidase Exhibits Wide Substrate Specificity. *Biochem. J.* **2001**, *358* (Pt 3), 607–614.
